# Investigations of the Chemical Composition and Aromatic Properties of Peel Essential Oils throughout the Complete Phase of Fruit Development for Two Cultivars of Sweet Orange (*Citrus sinensis* (L.) Osb.)

**DOI:** 10.3390/plants11202747

**Published:** 2022-10-17

**Authors:** Vincent Ferrer, Noémie Paymal, Carole Quinton, Félix Tomi, François Luro

**Affiliations:** 1 UMR AGAP Institut, University of Montpellier, CIRAD, INRAE, Institut Agro, 20230 San Giuliano, France; 2 Rémy Cointreau, Les Molières, 49124 Saint-Barthélemy-d’Anjou, France; 3 UMR SPE 6134, Université de Corse, CNRS, Equipe chimie et Biomasse, 20000 Ajaccio, France

**Keywords:** fruit maturity, fruit growth, sensorial analysis, oxygenated compounds, GC/MS, GC-FID

## Abstract

The peel essential oil (PEO) of sweet orange is used for flavoring liquors or foods and in the perfumery and cosmetics industry. The fruit maturity stage can modify the essential oil composition and aromatic properties, but little information is available on the evolution of PEO during the entire time set of fruit development. In this study, the yield, chemical composition and aromatic profile over the three phases of orange development were monitored. Four fruit traits (peel color, weight, acidity and sweetness) were recorded to characterize fruit development. Fruits of two sweet orange cultivars were sampled every two weeks from June to May of the next year. PEO was obtained by hydrodistillation and analyzed by gas chromatography coupled with a flame ionization detector (GC-FID). Compounds were identified with GC coupled with mass spectrometry (GC/MS). Ten expert panelists using the descriptor intensity method described the aromatic profile of PEO samples. The PEO composition was richer in oxygenated compounds at early fruit development stages, with an aromatic profile presenting greener notes. During fruit growth (Phases I and II), limonene’s proportion increased considerably as a few aliphatic aldehydes brought the characteristic of orange aroma. During fruit maturation (from November to March), the PEO composition and aromatic profile were relatively stable. Later, some modifications were observed. Regardless of the fruit development stage, the two sweet oranges presented distinct PEO compositions and aromatic profiles. These results constitute a temporal reference for the chemical and aromatic evolution of sweet orange PEO in the fruit development process under Mediterranean conditions. During the first two phases of fruit development, many changes occur in the PEO composition and aroma, suggesting that their exploitation could create new products.

## 1. Introduction

The fruit peel of sweet orange (*Citrus sinensis* (L.) Osb. or *C.* X. *aurantium* var *sinensis*) is a major source of aromatic extracts for perfumery, the agroindustry as a flavoring agent and for numerous minor uses [1]. The peel essential oil (PEO) of sweet orange is the most produced citrus oil, representing more than 60% of the total production [2]. The PEO of sweet orange has been reviewed a couple of times, and all studies have agreed that monoterpenes, mainly limonene, accounting for more than 95% of the total oil in mature fruit, dominate the sweet orange PEO [3]. The remaining compounds are predominantly aliphatic aldehydes at approximately one percent and sesquiterpenes at trace levels. Twenty-two to fifty-five essential oil compounds have been considered contributors to the sweet orange aroma [4,5,6]. Many factors, such as cultivar, fruit-ripening stage, geographic origin, extraction methods, storage conditions, postharvest treatment and health of the tree, can modify the citrus PEO composition [7,8,9,10,11,12,13,14,15,16,17,18,19,20,21].

A few studies have described the influence of seasonal variation on the PEO composition of citrus. Attaway et al. (1967) analyzed the PEO composition of “Dancy” mandarin, “Hamlin” orange and “Marsh” grapefruit at biweekly intervals from spring until harvest. The greatest changes were observed during fruit maturation, and they were characterized by a decrease in linalool, while the limonene concentration exhibited a corresponding increase [14]. Scora and Newman (1967) published a multisite comparison of the PEO composition of “Valencia Late” sweet orange using the sugar/acid ratio as an indicator of fruit maturity but only for three months during fruit maturation. The results did not permit the establishment of clear trends of the evolution of PEO components [22]. Coggins et al. (1999) described the evolution of the PEO composition from the early stages of the harvest period until the advanced stage of the harvest period. Linalool and geranial decreased with maturation progress, valencene increased and other compounds appeared stable [23]. This experiment also aimed to understand the influence of gibberellin treatment on fruit senescence, and the authors concluded that gibberellin delayed the evolution of the peel essential oil. Trozzi et al. (1999) observed an increase in monoterpene olefins (mainly limonene) during orange fruit maturation, whereas the proportion of alcohol monoterpenes and aliphatic compounds was reduced [24]. A recent study on PEO extracted by solvents analyzed the composition according to four stages, corresponding to significant changes in fruit size and color (50, 140, 200 and 230 days post-flowering) [25]. The majority of monoterpenes (α-pinene, limonene, linalool and α-terpineol), sesquiterpenes (valencene, α-sinensal and β-sinensal) and aliphatic aldehydes (octanal, decanal and dodecanal) increased between 50 and 140 days. The yield of PEO increased during sweet orange fruit growth to reach approximately 1.1 mL per fruit at maturity [26]. The PEO yield seems to be influenced by the year and by the cultivar [27,28].

Numerous publications on the PEO composition and yield of sweet oranges are available, but the main issue is the lack of information on the exact description of the fruit development stage, which makes any comparison between studies difficult [14,23,28]. Moreover, the fruits were defined as ripe or unripe without any chemical indicators of ripeness, or sometimes this state was only based on fruit color. Fruit color cannot be considered a standard indicator of fruit maturity because it is influenced by environmental factors, such as temperature and light [29,30]. Finally, comparison is often based on one sample or without statistical analysis, making numerous studies inconclusive.

The objective of this work is to provide a detailed study of the evolution of the PEO composition and yield at two week intervals during the entire fruit development process from fruitlets (2 months after anthesis) to the latest maturity stages (after harvest period). To describe the fruit development corresponding to PEO precisely, the evolution of the usual criteria of fruit development was recorded over the experimental period. The effect of PEO composition on the flavor profile was analyzed during fruit development with PEO samples representing the main periods of chemical variations.

## 2. Materials and Methods

### 2.1. Raw Material

Two cultivars of sweet orange (C. X. *aurantium* var. *sinensis* Blanco) were studied: “Cara Cara Navel” (SRA 666), with pink flesh, and “Madame Vinous” (SRA 551), with blond and seedy fruit. The fruits were harvested from five trees of each cultivar from 1 June 2019 to 15 June 2020. All biological materials used in this study came from the orchard of the INRAE-Cirad citrus collection at San Giuliano, France (latitude 42°17′ N, longitude 9°32′ E; Mediterranean climate, average rainfall of 840 mm per year and average temperature of 15.2 °C; soil derived from alluvial deposits and classified as fersiallitic, with a pH range of 5.0–5.6). All trees were grafted onto the same rootstock and Carrizo citrange [*C. sinensis* X *Poncirus trifoliata*] and grown under identical conditions [31].

### 2.2. Phenological Parameters

Every two weeks during the experiment, five fruits per tree (x five trees) were randomly picked for each cultivar and described by the following parameters: fruit weight, peel color, acidity and sweetness of the juice. Flavedo color was evaluated by the citrus color index (CCI) with L, a* and b* determined with the CHROMA METER CR-400, (Konica Minolta Sensing, Ramsey, NJ, USA) [32]. The color indices of each fruit were determined by measuring four equidistant positions around the fruit’s equatorial line.

Juicy fruits were separately hand-pressed, and the total soluble solid (TSS) content expressed in °Brix was measured using an RFM710 refractometer (Bellingham + Stanley^®^, Weilheim in Oberbayern, Germany). The titratable acidity (AC) expressed as a percentage (g of citric acid per 100 g of juice) was measured using an 855 Robotic Titrosampler (ΩMetrohm^®^, Bâle, Switzerland). At each harvesting date, three fruits from each tree were harvested and the peel dry matter percentage was calculated using an oven until the weight of all samples stabilized. This peel dry matter was then used to calculate the yield of peel essential oil (PEOY).

### 2.3. Peel Oil Analysis

#### 2.3.1. Hydrodistillation

Once the phenological parameters were measured, the fruits were hand-peeled using a knife, and the peel was stored at −20 °C before further analysis. All processes were performed in less than a day. Before hydrodistillation, the frozen fresh peel material picked every 15 days all along the experience was blended with distilled water for one minute using a Model 1300 W blender (Magimix^®^, Vincennes, France) to disrupt the cells of PEO. Grounded peels were introduced in a 2 L wide neck flask reaction completed with distilled water to 1 L and then heated two and a half hours using a heating mantle Model EM2000/CE (Electrothermal^®^, London, UK). The PEO was obtained using a Clevenger apparatus, cooled using a refrigerated fluid (mix of glycol/water) at 4 °C and moved by a minichiller Model C20 (Huber^®^, Offenburg, Germany). The total PEO was collected and weighed. Then, the PEOs were placed in overfull 300 µL tainted vials and stored at −20 °C before further analysis.

#### 2.3.2. GC (FID) and GC/MS Analyses

Gas chromatography analyses were performed on a Clarus 500 gas chromatograph (PerkinElmer, Waltham, MA, USA) equipped with a flame ionization detector and two fused silica gel capillary columns (50 m, 22 mm i.d., film thickness 0.25 μm), BP-1 (polydimethylsiloxane) and BP-20 (polyethylene glycol). The oven temperature was programmed from 60 to 220 °C at 2 °C/min and then held isothermally at 220 °C for 20 min, with an injector temperature of 250 °C, detector temperature of 250 °C, carrier gas of hydrogen (1.0 mL/min), and a split of 1/60. The relative proportions of components were expressed as percentages, obtained by peak area normalization. Retention indices were determined relative to the retention times of a series of *n*-alkanes (C7–C28) with linear interpolation (“Target Compounds” software of Perkin Elmer).

Gas chromatography coupled with mass spectrometry was conducted with a TurboMass quadrupole detector (Perkin Elmer, Waltham, MA, USA) directly coupled to an Autosystem XL (Perkin Elmer) equipped with a fused silica gel capillary column (50 m, 0.22 mm id, film thickness 0.25 μM) (BP-1 polydimethylsiloxane). Carrier gas was helium at 0.8 mL/min, split was 1/75, injection volume was 0.5 μL, injector temperature was 250 °C, energy ionization was 70 eV and electron ionization mass spectra were acquired in the data. For further information, refer to the publication by Luro et al. [33].

#### 2.3.3. Sensorial Analysis

The panel was composed of 10 expert panelists (four men aged between 24 and 42 with an average age of 33 and six women aged between 23 and 54 with an average age of 43) familiarized with sensorial analysis of citrus peel essential oils. Only the samples from the beginning of each month were used for the sensorial analysis (1 July, 3 August, 1 September, 1 October, 1 November, 1 December, 7 January, 3 February and 3 March).

First, a free sorting task was conducted on the nine samples for each cultivar. The closest samples were merged in equal proportion to five final clusters per cultivar: July (Jul), August to September (Aug–Sep) October to November (Oct–Nov), December to January (Dec–Jan) and February to March (Feb–Mar) for “Cara Cara Navel” sweet orange; and July (Jul), August to October (Aug–Oct), November to January (Nov–Jan), February (Feb) and March (Mar) for “Madame Vinous” sweet orange.

Lastly, a session was released for each cultivar using the gathered samples. The samples were described with the most relevant descriptors unanimously chosen by panelists. During consensus sessions, six descriptors of aroma were defined for the two sweet oranges. The value of each descriptor ranged from 0 (absence) to 5 (highest intensity of the descriptor). The aromatic profiles of PEO of the two sweet oranges were different; therefore, different descriptors were used (Table 1).

Panelists, isolated from each other, analyzed the samples conditioned into indistinguishable tubes in a randomized order. The panelists had as much time as they wanted to rate all descriptors. Two or three samples were analyzed by session to avoid weariness and desensitization. The experiment was conducted over a couple of weeks.

#### 2.3.4. Statistical Analysis

Analysis of variance (ANOVA) followed by Tukey’s test was conducted on the PEO weight per fruit (EOW) and per 100 g of fruit (EOY) to determine the influence of the development stage on these parameters using the ”agricolae” package in “R” software. The same package and the same function were used to determine the existence of a significant difference within each aromatic descriptor during the development of the fruit [34].

The evolution of the PEO composition was represented with principal component analysis (PCA) using the package “Factoextra” in R [35].

The differences in PEO composition between the two sweet orange cultivars were represented on a stacked histogram by the ratio of the difference between proportions of each cultivar divided by the sum of the proportions of the two cultivars. Statistical differences between compounds of both cultivars were calculated using the Kruskal-Wallis test (*p* < 0.05) using the “stats” package in R [36].

The correlation between aromatic descriptors and compounds was calculated using the partial least square regression method. The results represented a circle of correlation for sweet orange cultivars and a heatmap for sour orange using the “mixOmics” package in R [37].

## 3. Results

### 3.1. Description of Fruit Development

**Figure 1:**
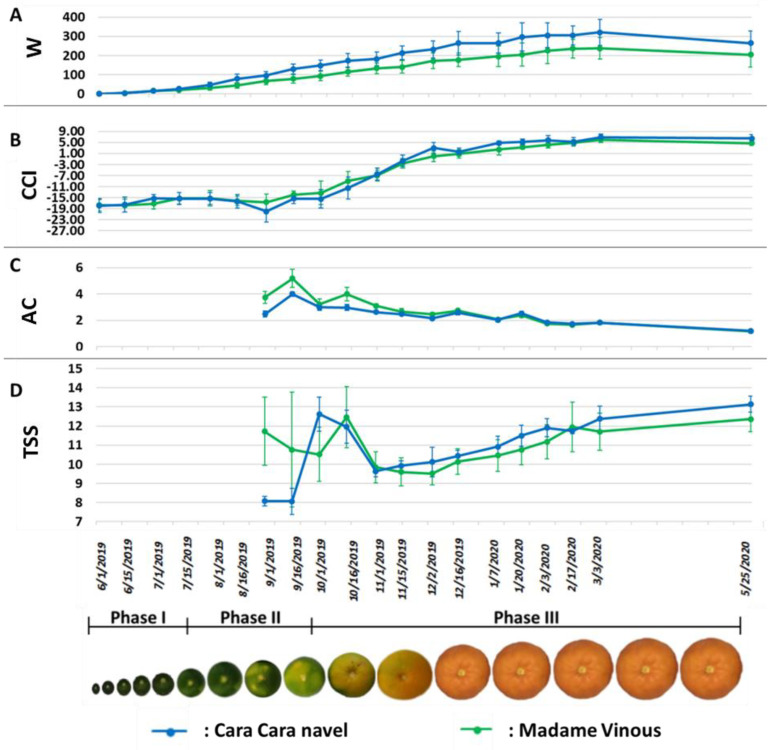
Dot plots representing the evolution of phenological parameters at different dates of fruit development. The vertical bar on each dot represents the standard deviation. (**A**) fruit weight (W) (in g); (**B**) citrus color index (CCI) of the flavedo; (**C**) titrated acidity (AC) expressed in % of citric acid; and (**D**) total soluble solid (TSS) content expressed in °Brix.

The phenological parameters evolved quite similarly for the two sweet orange cultivars during fruit development (Figure 1). The average fruit weight increased continuously from the end of July until the end of February (Figure 1A). After this date, the weight seemed to decrease slightly but was not confirmed by statistics due to the variation between fruits. The flavedo color (CCI) displayed a sigmoidal profile (Figure 1B). Until the end of September, the fruit rind was dark green; then, in October, the CCI started to increase, corresponding to the degreening process, reaching positive values at the end of November, indicating the appearance of a light orange color. The maximum CCI values were reached in February and remained stable thereafter. The correlation between the CCI value and the fruit external color can be visually appreciated by fruit photography at the bottom of Figure 1.

Values for acidity (AC) and total soluble solid (TSS) content were obtained only in September and not before because it was not possible to extract the pulp juice (Figure 1C,D). The juice acidity peaked in mid-September and then decreased slowly to less than 2% at the beginning of December and later approached 1% in mid-June. TSS fluctuated greatly during the first stages of fruit development for both cultivars. From November to the end of the experiment, the TSS increased similarly for the sweet orange cultivars, reaching 12°Brix in February.

According to Bain (1958) and Iglesias et al. (2007), by considering the evolution of fruit weight and the acidity of fruit juice, the three phases of fruit development can be dated. Phase I corresponding to cellular multiplication stopped when the increase in fruit weight started (mid-July) [38,39]. This increase in fruit weight corresponded to the entry of water into the pulp cells and the beginning of Phase II or phase of cellular enlargement. At this time, the biosynthesis of sugar and organic acids started [40]. Phase III corresponding to fruit maturation began when acidity decreased (in September). There was also the date of skin color change under the Mediterranean climate. The period of these three phases of fruit development are specified at the bottom of the figures to show when the changes in characters occurred.

### 3.2. Evolution of PEO Yield during Fruit Development

**Figure 2:**
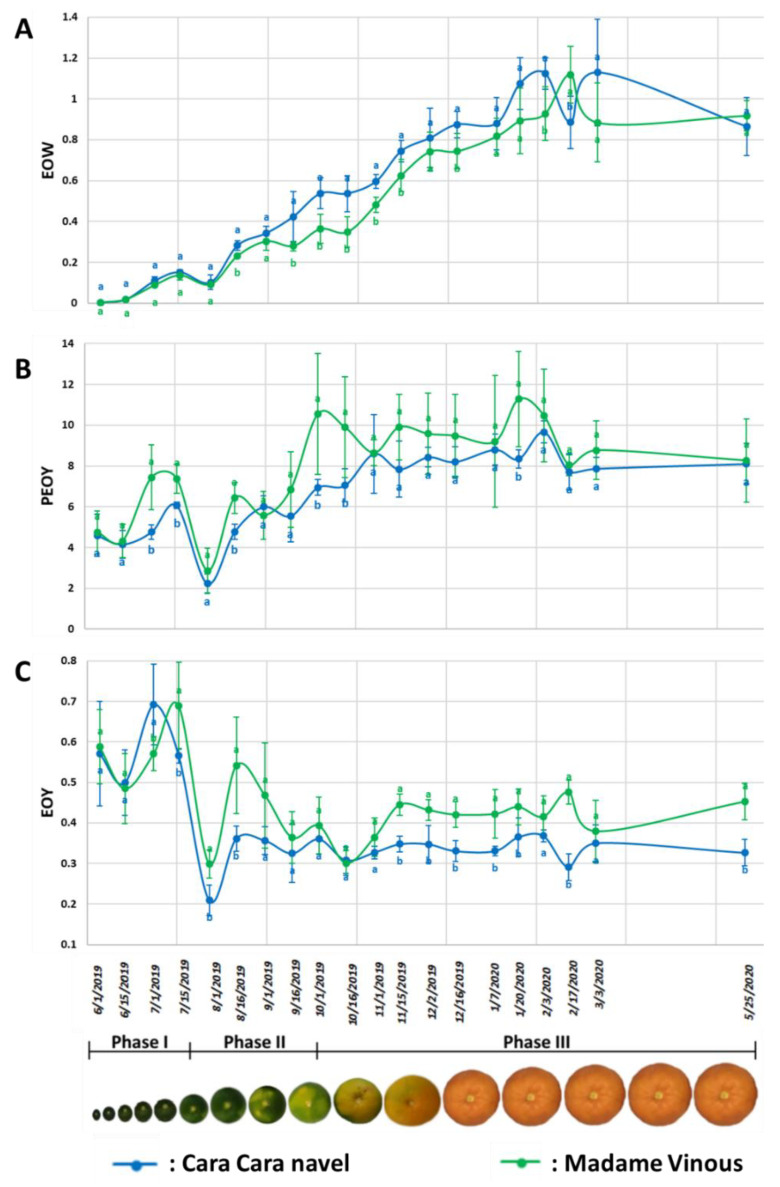
Dot plots representing the evolution of the average weight (g) of essential oil per fruit (**A**), the average weight (g) of essential oil per 100 g of dried peel (**B**) and the average weight (g) of essential oil per 100 g of fresh fruit (**C**) throughout fruit development. The vertical bar represents the standard deviation, and the letter in each plot represents the statistical group according to Tukey’s test (*p* ≤ 0.05) of differences between cultivars at each date.

The weight of PEO per fruit (EOW) evolved similarly to the weight of the fruit (W), constantly increasing from June to January and then stabilizing when it reached its maximum value (Figure 2A). For the two cultivars, the maximum EOW per fruit was approximately 1 g. The yield of PEO (PEOY) expressed in g per 100 g of dried peel increased at the beginning of Phase III and reached a maximum value slightly higher than 8 g per 100 g of dried peel (Figure 2B). The weight of PEO per 100 g of fresh fruit (EOY) was higher during Phase I, slightly decreased during Phase II and stabilized in the maturation phase (Figure 2C). The EOY stabilized at the beginning of November and remained constant later. The EOY of “Madame Vinous” sweet orange was slightly higher than that of “Cara Cara Navel”, with values of 0.42% and 0.35%, respectively (main value calculated with the last 10 samples).

### 3.3. Evolution of the PEO Chemical Composition during Fruit Development

**Figure 3:**
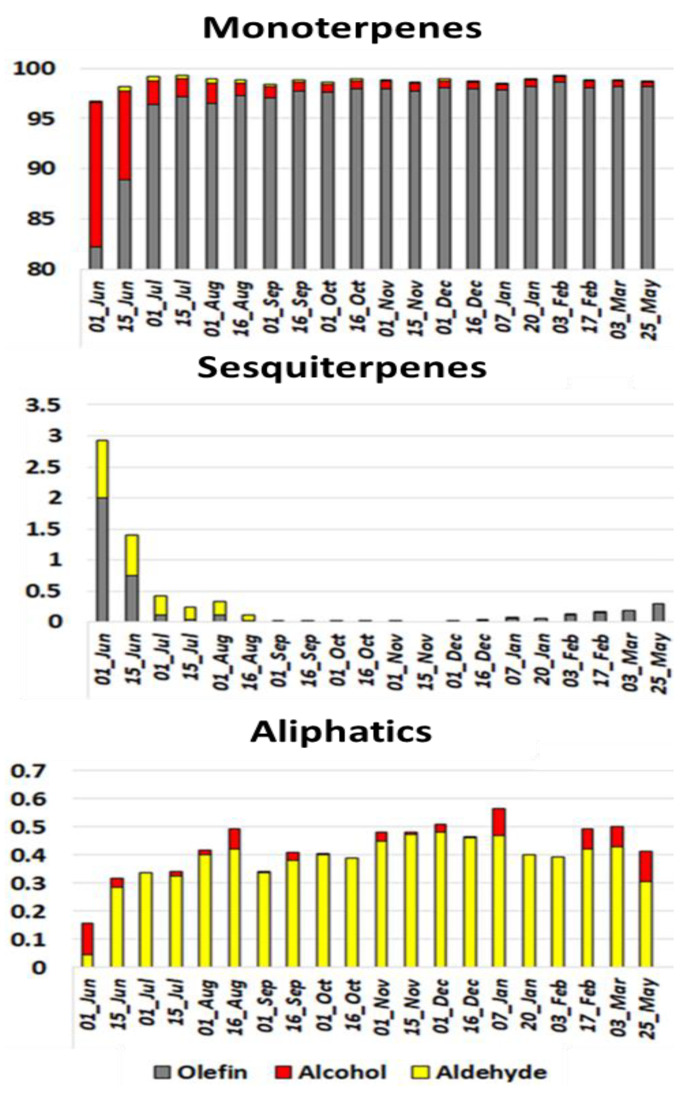
Stacked bar plot representing the mean proportion of major chemical families of the “Cara Cara navel” sweet orange cultivar during fruit development.

**Figure 4:**
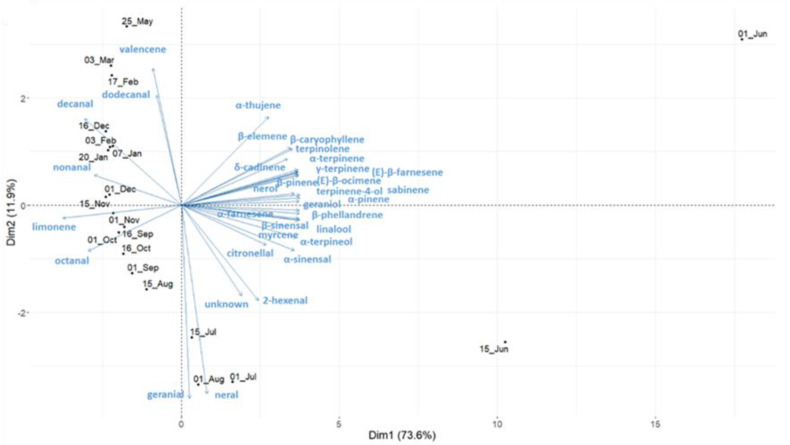
Biplots of principal component analysis representing the diversity of the PEO composition of each sampling date (in black) and the contribution of the 33 main aromatic compounds (in blue). Each sampling date represents the average composition of each compound of the five repetitions of “Cara Cara Navel” sweet orange.

The overall composition of the two sweet orange cultivars evolved similarly during ripening, and sixty-five and sixty-nine compounds were identified during the development cycle for “Cara Cara navel” and “Madame Vinous”, respectively (Table S1). Monoterpenes dominated the global composition at all stages, but major changes occurred in the earliest stages, particularly at Phase I of fruit development (Figure 3). During Phase I, the proportion of almost all monoterpenes decreased, whereas the proportion of limonene increased considerably from 25 to 95%. Sabinene, linalool and α-terpineol decreased greatly from 38%, 7% and 6% to reach 0.6% for linalool and traces for the two others, respectively. Among monoterpenes, only neral and geranial followed a different trend and peaked between July and August. This monoterpene family was the best contributor to the date sample diversity on the second axis of the PCA (Figure 4), where the chemical compositions from June to August were clearly distant from other samples.

The proportion of sesquiterpenes evolved mostly in the first two phases of fruit development (June to August). α-sinensal, β-sinensal, β-caryophyllene, (E)-β-farnesene, β-elemene and α-farnesene were all approximately 0.5% at the first sampling date and decreased rapidly to be undetected or detected in traces in September. The only sesquiterpene that followed a different trend was valencene, which appeared at advanced fruit maturity and increased to 0.25% at the last sampling date. Sesquiterpenes were the major contributors to the date sample diversity represented by the first axis of the PCA (Figure 4). The chemical profiles of fruitlets harvested in June were clearly atypical from all other date samples, with the greatest variation in monoterpene and sesquiterpene composition. The samples from the other dates were distributed sequentially, almost linearly, along PCA Axis 2.

Aliphatic aldehydes remained globally constant, but each compound peaked at different times (Figure 4). The longer the carbon chains were, the later they peaked. Finally, aliphatic alcohols remained in trace amounts during the whole development cycle.

### 3.4. Differences in PEO Chemical Composition between Sweet Orange Cultivars

**Figure 5:**
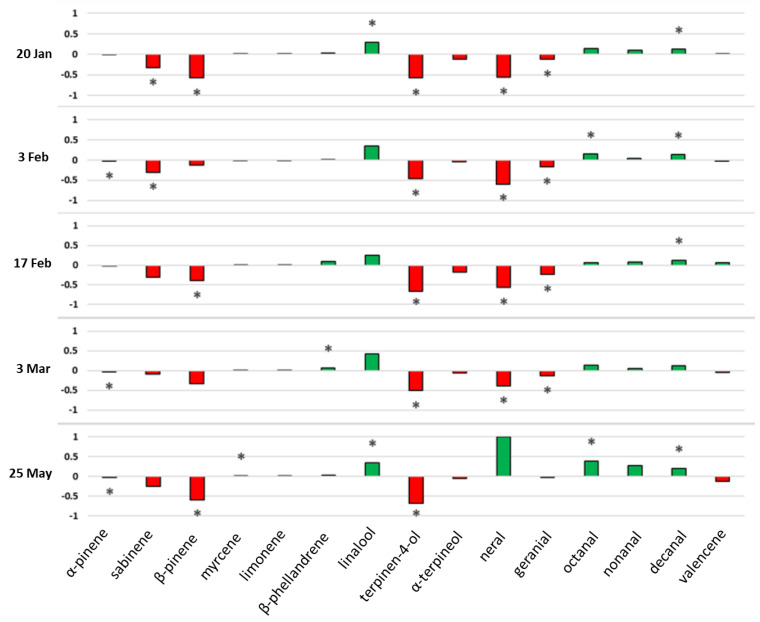
Stacked histogram representing the evolution of the 15 main compounds between January and May for the PEO of the two cultivars. When the proportion of a compound in “Cara Cara Navel” was superior to that in “Madame Vinous”, the bar is red, and in the opposite situation, the bar is green. *—Significant differences between the two varieties. The asterisk indicates a significant difference with the Kruskal-Wallis test (*p* < 0.05).

Although the chemical compositions of both sweet oranges were similar, some significant differences were detected at the late maturation stage when the PEO composition was the most stable (Figure 5).

The proportions of α-pinene, sabinene, β-pinene, terpinen-4-ol, α-terpineol and geranial were always superior in “Cara Cara Navel”. The proportions of β-phellandrene, linalool, octanal, nonanal and decanal were always superior in “Madame Vinous” regardless of the date of analysis. Neral was four times higher in “Cara Cara Navel” than in “Madame Vinous” but the ratio is inversed on 25 May because neral was undetected in “Cara Cara Navel” at this date.

### 3.5. Aromatic Profile during Fruit Development

**Figure 6:**
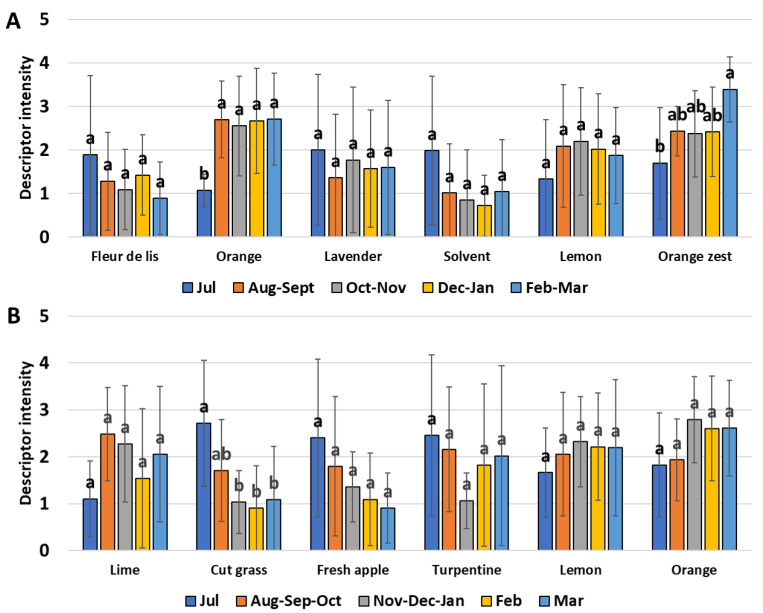
Bar plots representing the evolution of the five sensory descriptors of “Cara Cara Navel” (**A**) and “Madame Vinous” (**B**) sweet oranges during fruit development. The vertical bar represents the standard deviation, and the letter represents the statistical group according to Tukey’s test.

Only two descriptors (“Orange” and “Orange zest”) were detected as significantly different between dates for the “Cara Cara Navel” cultivar (Figure 6A). The intensity of the “Orange” descriptor was the lowest in the earliest sampling date (July) and stronger in more advanced fruit maturity stages. The “Orange zest” descriptor was lowest in July and highest between February–March. The four other descriptors (“Fleur de lis”, “Lavender”, “Solvent” and “Lemon”) were considered statically equivalent.

The “Cut grass” character was the only descriptor that was significantly different between the sampling dates for the “Madame Vinous” cultivar (Figure 6B). Its intensity was maximum in July and minimum from November to March. All other descriptors (“Lime”, “Fresh apple”, “Turpentine”, “Lemon” and “Orange zest”) were statistically equivalent between sampling dates.

### 3.6. Correlation between Aromatic Profile and PEO Composition

**Figure 7:**
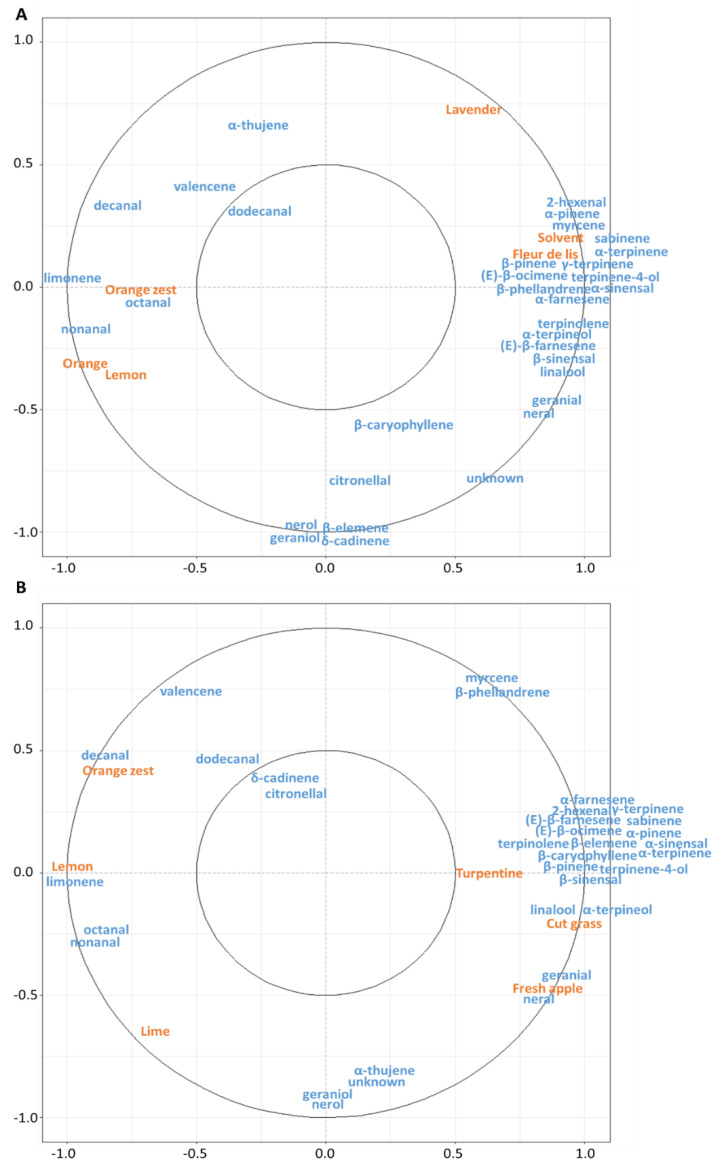
Correlation circles between PEO compounds and aromatic descriptors using partial least squares regression. Compounds are represented in blue, and aromatic descriptors are represented in orange. The inner circle corresponds to a correlation of (±) 0.5, and the outer circle corresponds to (±) 1. (**A**) “Cara Cara Navel” and (**B**) “Madame Vinous”.

The “Orange zest” and “Lemon” descriptors correlated with the same compounds in both cultivars, notably with limonene, octanal, nonanal, decanal, dodecanal and valencene (Figure 7). Nerol, geraniol, δ-cadinene, citronellal, and α-thujene did not correlate with any sensory descriptors in the two cultivars.

In the PEO of the “Cara Cara Navel” cultivar, the descriptor “Orange” was highly correlated with “Lemon” and “Orange zest” (Figure 7A). The “Solvent” and “Fleur de lis” descriptors were highly correlated with seventeen compounds that were present in higher concentrations at the earliest stage of fruit development. The “Lavender” descriptor did not correlate with any compounds. Neral, geranial, β-caryophyllene and β-elemene were not correlated with any descriptor.

Other correlations were established in the PEO of “Madame Vinous” (Figure 7B). The “Fresh apple” descriptor correlated with neral and geranial. The “Lime” descriptor did not correlate clearly with any compound. Linalool and α-terpineol correlated strongly with the “Cut grass” descriptor and less strongly with the “Turpentine” descriptor. These two descriptors also correlated with sixteen other compounds. Myrcene and β-phellandrene were not correlated to any descriptor.

## 4. Discussion

### 4.1. Orange Fruit Development

The evolution of the fruit quality parameters (soluble sugar content and acidity) as well as the fruit mass was in accordance with the observations already made on the development and ripening of oranges [39,40]. The only deviation from expectations was in the evolution of soluble sugar content during Phase II (the cell growth phase), which did not follow the sigmoidal curve representative of this developmental phase of all citrus fruits, with a rapid increase in sugar content and then a slowdown at the beginning of Phase III [39,40]. The decrease in acidity during Phase III depends on the species (marked in oranges, mandarins and clementines) and the variety whose maturity range depends on the speed of this decrease. The TSS chaotic evolution during Phase II of the two sweet oranges was probably because it was measured with a refractometer, which requires prior extraction of the juice, and in September, the fruits still had a low percentage of juice. The peak of acidity was on 16 September and should then have gradually decreased. In our experiment, the value on 1st October appeared abnormally lower than the following points. It is possible that this variation in acidity was linked to dilution following intense rainfall in the previous days. Acidity is more sensitive if a massive water supply arrives after a long period of summer drought despite irrigation [41]. The color change of the orange peel occurred at the end of Phase II (October) by the beginning of degreening (CCI increases), and then the orange color appeared in mid-November (CCI became positive). The variation in fruit color in trees depends on atmospheric temperature and light exposure [29,30]. It is clear that in areas where the temperature remains constant or does not decrease sufficiently, the fruits remain green. Therefore, color cannot be used as a criterion to describe the evolution of fruit maturity in all climatic conditions.

Under Corsican growing conditions, these two sweet orange cultivars are considered ripe when the acidity is approximately 2% and the TSS exceeds 11° Brix. These conditions were reached at the beginning of January, while the skin orange color was acquired 1 month earlier.

The fruit weight evolved similarly for the two cultivars during fruit development by a sigmoidal flat curve, as described in all citrus fruit species [26,39]. This parameter seems to be less dependent on environmental conditions under regular watered and fertilized growth control than other fruit quality parameters [42]. This finding, the association between the evolution of PEO composition and the linear weight increases between October and the end of February, suggests the use of fruit weight as an indicator of fruit development status no matter where it is grown.

### 4.2. The PEO Yield

The two sweet orange cultivars produced a similar PEO yield. These results are in accordance with the literature; indeed, a difference in PEO yield between cultivars has already been observed between sweet orange cultivars and over the years [26,27,28]. The essential oil weight per fruit (EOW) is known to be dependent on the environment [26]. However, the value observed in Corsica is in accordance with observations in other countries [26,27,43,44]. The peel essential oil yield (PEOY), expressed as relative to dried peel weight, increased from June to October and stabilized later on. This observation suggests a multiplication of EO vesicles during this period of fruit enlargement or an increase in EO synthesis during this phase but not an increase in EO vesicle density. Finally, the essential oil weight/100 g of fresh fruit (EOY) was higher in immature fruits, which is in agreement with the higher proportion of skin in fruitlets than in mature fruits [45].

### 4.3. PEO Chemical Composition and Aromatic Profile of Sweet Oranges

Compounds, such as limonene/linalool oxide, carvone, and carveol, were identified as products of degradation [7,46,47]. They were removed from the statistical analysis.

The PEO composition of both sweet orange cultivars evolved similarly and was dominated by monoterpenes, with low amounts of aliphatics and sesquiterpenes in traces at an advanced maturity stage, as previously observed by other authors [3,48].

PEOs from June to August were richer and more varied in monoterpenes and sesquiterpenes, with significantly more oxygenated mono- and sesquiterpenes than on all other sample dates. Although this result was in agreement with Coggins et al. [23] limonene, neral and geranial were the only monoterpenes in higher proportions at more advanced stages in our study. The evolution of limonene neral and geranial has already been observed [14,23,25]. Among aliphatics, clear trends were identified; octanal, nonanal, decanal and dodecanal appeared in this order, and their proportions increased until fruit maturity, as previously observed [25]. Finally, a single sesquiterpene, valencene, appeared in mid-December and increased significantly in proportion as the fruit matured. Valencene was described as an indicator of advanced fruit maturity [23,25].

These variations in chemical composition were associated with profound changes in aromatic profiles. First, the PEO aromatic profiles of the two sweet orange cultivars were considered sufficiently different to have only two descriptors in common (“Lemon” and “Orange zest”). At first glance, the standard deviation is relatively high, compared to the mean, indicating a contrast of perception in the descriptor’s intensities between the ten judges. Limonene, octanal, nonanal decanal, dodecanal and valencene were correlated with the intensity of the “Lemon”, “Orange”, “Orange zest”, and “Lime”. Octanal, nonanal and decanal were identified in a previous GC-O study to have strong aroma intensity and may be important contributors to the mature orange aroma in peel or juice essential oil [4,5,6,49,50,51,52,53,54,55]. The aromatic activity of valencene remains unclear and controversial but is probably low or null [5,6,55]. Limonene has been identified as an active aroma compound but with a debated contribution to the mature orange aroma [5,6,49,50,51,52,53,54,56,57,58,59]. However, this compound is ultra-dominant in sweet orange PEO, and its role may not only be aromatic; some authors have suggested that it may act as a “lifting agent” for other volatiles [45]. Rodríguez et al. (2017), using transgenic sweet orange with down-regulated limonene synthesis, observed that a decrease in limonene content (51 times lower than that in regular orange) had no impact on the odor perception of orange juice, suggesting a low impact on the sensory profile [60]. In the “Madame Vinous” sweet orange cultivar, the descriptor “Fresh apple” correlated strongly with the two isomers, neral and geranial. This result is consistent with previous studies in which these compounds were identified as aromatic and had lemon/citrus/minty notes [4,5,6,53,56,57]. Limonene, linalool, octanal, decanal, dodecanal, geranial, neral, myrcene, α-sinensal, β-sinensal and citronellal were identified in a reconstruction study as the most important compounds to duplicate the aroma of navel orange [57].

Many compounds correlating with sensory descriptors were more intense in the earliest stage (“Solvent”, “Fleur de lis”, “Cut grass”, and “Turpentine”). This is explained by the fact that all these compounds were in higher proportions in fruit in Phase I of fruit development and decreased during Phase III. However, the proportions decreased and remained low, and they could still play a major role in the mature orange aroma. Among these compounds, the majority were identified as contributors to the sweet orange aroma, such as linalool, α-pinene, β-pinene, β-phellandrene, γ-terpinene, terpinolene, α-terpineol, terpinen-4-ol, β-caryophyllene, perillaldehyde, β-elemene and α-sinensal a,d β-sinensal [5,6,49,50,51,52,53,54,57,58,59,60].

### 4.4. Comparison of the Two Sweet Orange Cultivars

PEO compositions were only studied at advanced fruit maturity, avoiding the high variance between samples of the same date on the earliest dates not suitable to make any statistical comparison. The PEO comparison of different sweet orange cultivars at different maturity stages is relatively new. Oxygenated compounds (monoterpene alcohols and aliphatic aldehydes) differentiated the two sweet orange cultivars. Similar differences between “Navel” and “Blond” sweet orange types have already been observed [8,24]. Dugo et al. (1994) also identified differences in the PEO composition between “blond” and “blood” sweet orange types [61]. The differences between the “Cara Cara Navel” and “Madame Vinous” cultivars may lead to distinct aromatic profiles, and thus explain the choice of different aromatic descriptors made by panelists [4]. However, it is also plausible that compounds under the detection threshold of the GC are specific to a cultivar and provide a specific aromatic note, such as that in blood orange juice [59,62]. The low variation in PEO composition between oranges is a consequence of genetic diversification based on somatic mutations selected by growers that induce very restricted modifications of the genome sequence [63].

## 5. Conclusions

The present work is the most detailed description of the PEO yield, composition and aromatic profile of sweet oranges during fruit development from a few weeks after anthesis to the fruit abscission period. In addition, the PEO traits were anchored with the fruit physiological development described by the usual fruit quality parameters. They should be useful for comparison with other studies on sweet orange made in other countries. Fruit enlargement or weight seems to be the most suitable due to the lower impact of the environment on its evolution than acidity, sugar content or skin color. The main class, monoterpene olefins, increased significantly between the fruitlets and mature fruits, whereas most oxygenated monoterpenes decreased. The proportion of most sesquiterpenes decreased in the maturation phase, except valencene, which increased during this period. Octanal and decanal (aliphatic compounds) peaked at advanced fruit maturity. The intense “orange” and “fruity” notes for each cultivar increased when fruit maturation was occurring, whereas more intense “green” notes characterized the PEO of fruitlets.

## Figures and Tables

**Table 1:** Aromatic descriptors of sweet orange PEO and their descriptions.

Cultivar	Aromatic Descriptors	Description
‘‘Cara Cara Navel’’	Fleur de Lis	white flower, intense, heady
	Lavender	powerful, intense, floral
	Lemon	fruity, gourmet, fresh
	Orange	fruity, orange juice
	Orange zest	fruity, orange albedo, zesty
	Solvent	defect
‘‘Madame Vinous’’	Cut grass	green, vegetable
	Fresh apple	fresh, fruity
	Lemon	fruity, gourmet, fresh
	Lime	lemon, very zesty, very fresh, fruity
	Orange	fruity, orange juice
	Turpentine	vegetable, fresh, aromatic plant

## Supplementary Materials

The following supporting information can be downloaded at: https://www.mdpi.com/article/10.3390/plants11202747/s1. Table S1: PEO composition.

## References

[1] Kaur H., Singh G. (2021). Recent trends in citrus (*citrus* spp.) peel utilization: A review. Plant Arch..

[2] Dugo G., Di Giacomo A. (2002). Medicinal and aromatic plants industrial profiles. Citrus: The Genus Citrus.

[3] González-Mas M.C., Rambla J.L., López-Gresa M.P., Blázquez M.A., Granell A. (2019). Volatile Compounds in Citrus Essential Oils: A Comprehensive Review. Front. Plant Sci..

[4] Gaffney B., Havekotte M., Jacobs B., Costa L. CharmAnalysis of two citrus sinensis peel oil volatiles. Proceedings of the International Citrus Symposium.

[5] Högnadóttir Á., Rouseff R.L. (2003). Identification of Aroma Active Compounds in Orange Essence Oil Using Gas Chromatography–Olfactometry and Gas Chromatography–Mass Spectrometry. J. Chromatogr. A.

[6] Qiao Y., Xie B., Zhang Y., Zhang Y., Fan G., Yao X., Pan S. (2008). Characterization of Aroma Active Compounds in Fruit Juice and Peel Oil of Jinchen Sweet Orange Fruit (*Citrus sinensis* (L.) Osbeck) by GC-MS and GC-O. Molecules.

[7] Gaff M., Esteban-Decloux M., Giampaoli P. (2020). Bitter Orange Peel Essential Oil: A Review of the Different Factors and Chemical Reactions Influencing Its Composition. Flavour Fragr. J..

[8] Verzera A., Trozzi A., d’Alcontres I.S., Cotroneo A. (1996). On the Genuineness of Citrus Essential Oils. Part XLVIII. The Composition of Volatile Fraction of Some Varieties of Sweet Orange Oils. J. Essent. Oil Res..

[9] Sawamura M., Tu N.T.M., Yu X., Xu B. (2005). Volatile Constituents of the Peel Oils of Several Sweet Oranges in China. J. Essent. Oil Res..

[10] Xiao Z., Ma S., Niu Y., Chen F., Yu D. (2016). Characterization of Odour-Active Compounds of Sweet Orange Essential Oils of Different Regions by Gas Chromatography-Mass Spectrometry, Gas Chromatography-Olfactometry and Their Correlation with Sensory Attributes: Characterization of Odor-Active Compounds of Sweet Orange Oils. Flavour Fragr. J..

[11] Zouaghi G., Najar A., Aydi A., Claumann C.A., Zibetti A.W., Ben Mahmoud K., Jemmali A., Bleton J., Moussa F., Abderrabba M. (2019). Essential oil components of *citrus* cultivar ‘maltaise demi sanguine’ (*Citrus sinensis*) as affected by the effects of rootstocks and viroid infection. Int. J. Food Prop..

[12] Njoroge S.M., Phi N.T.L., Sawamura M. (2009). Chemical Composition of Peel Essential Oils of Sweet Oranges (*Citrus Sinensis*) from Uganda and Rwanda. J. Essent. Oil Bear. Plants.

[13] Luro F., Garcia Neves C., Costantino G., da Silva Gesteira A., Paoli M., Ollitrault P., Tomi F., Micheli F., Gibernau M. (2020). Effect of Environmental Conditions on the Yield of Peel and Composition of Essential Oils from Citrus Cultivated in Bahia (Brazil) and Corsica (France). Agronomy.

[14] Attaway J., Pieringer A., Barabas L. (1967). A study of the percentage variations in peel and leaf oil terpenes during one season. Phytochemistry.

[15] Verzera A., Trozzi A., Dugo G., Di Bella G., Cotroneo A. (2004). Biological Lemon and Sweet Orange Essential Oil Composition. Flavour Fragr. J..

[16] Xu B.M., Baker G.L., Sarnoski P.J., Goodrich-Schneider R.M. (2017). A Comparison of the Volatile Components of Cold Pressed Hamlin and Valencia (*Citrus sinensis* (L.) Osbeck) Orange Oils Affected by Huanglongbing. J. Food Qual..

[17] Sun X., Yang H., Zhao W., Bourcier E., Baldwin E.A., Plotto A., Irey M., Bai J. (2021). Huanglongbing and Foliar Spray Programs Affect the Chemical Profile of “Valencia” Orange Peel Oil. Front. Plant Sci..

[18] Benjamin G., Tietel Z., Porat R. (2013). Effects of Rootstock/Scion Combinations on the Flavor of Citrus Fruit. J. Agric. Food Chem..

[19] Usai M., Arras G., Fronteddu F. (1992). Effects of Cold Storage on Essential Oils of Peel of Thompson Navel Oranges. J. Agric. Food Chem..

[20] Ferhat M.A., Meklati B.Y., Chemat F. (2007). Comparison of Different Isolation Methods of Essential Oil FromCitrus Fruits: Cold Pressing, Hydrodistillation and Microwave ‘Dry’ Distillation. Flavour Fragr. J..

[21] Farahmandfar R., Tirgarian B., Dehghan B., Nemati A. (2020). Changes in Chemical Composition and Biological Activity of Essential Oil from Thomson Navel Orange (*Citrus Sinensis* L. Osbeck) Peel under Freezing, Convective, Vacuum, and Microwave Drying Methods. Food Sci. Nutr..

[22] Scora R.W., Newman J.E. (1967). A phenological study on the essential oils of the peel of Valencia oranges. Agric. Meteorol..

[23] Coggins C.W., Scora R.W., Lewis L.N., Knapp J.C.F. (1969). Gibberellin-Delayed Senescence and Essential Oil Changes in the Navel Orange Rind. J. Agric. Food Chem..

[24] Trozzi A., Verzera A., Lamonica G. (1999). Essential Oil Composition of *Citrus Sinensis* (L.) Osbeck Cv. Maltese. J. Essent. Oil Res..

[25] Goh R.M.V., Pua A., Ee K.H., Huang Y., Liu S.Q., Lassabliere B., Yu B. (2021). Investigation of Changes in Non-Traditional Indices of Maturation in Navel Orange Peel and Juice Using GC–MS and LC-QTOF/MS. Food Res. Int..

[26] Bartholomew E.T., Sinclair W.B. (1946). Factors influencing the volatile oil content of the peel of immature and mature oranges. Plant Physiol..

[27] Wolford R.W., Kesterson J.W., Attaway J.A. (1971). Physicochemical Properties of Citrus Essential Oils from Florida. J. Agric. Food Chem..

[28] Kesterson J.W., Braddock R.J. (1975). Total peel oil content of the major florida citrus cultivars. J. Food Sci..

[29] Carmona L., Zacarías L., Rodrigo M.J. (2012). Stimulation of Coloration and Carotenoid Biosynthesis during Postharvest Storage of ‘Navelina’ Orange Fruit at 12 °C. Postharvest Biol. Technol..

[30] Lado J., Alós E., Manzi M., Cronje P.J.R., Gómez-Cadenas A., Rodrigo M.J., Zacarías L. (2019). Light Regulation of Carotenoid Biosynthesis in the Peel of Mandarin and Sweet Orange Fruits. Front. Plant Sci..

[31] Luro F., Bloquel E., Tomu B., Costantino G., Tur I., Riolacci S., Varamo F., Ollitrault P., Froelicher Y., Curk F. (2017). The INRACIRAD Citrus Germplasm Collection of San Giuliano, Corsica.

[32] Jimenez-Cuesta M., Cuquerella J., Martinez-Javaga J.M. Determination of a color index for citrus fruit de-greening. Proceedings of the International Society of Citriculture/International Citrus Congres.

[33] Luro F., Viglietti G., Marchi E., Costantino G., Scarpa G.M., Tomi F., Paoli M., Curk F., Ollitrault P. (2019). Genetic, Morphological and Chemical Investigations Reveal the Genetic Origin of Pompia (C. Medica Tuberosa Risso & Poiteau)—An Old Endemic Sardinian Citrus Fruit. Phytochemistry.

[34] Gomez K.A., Gomez A.A. (1984). Statistical Procedures for Agricultural Research.

[35] Kassambara A., Mundt F., Factoextra: Extract and Visualize the Results of Multivariate Data Analyses (2020). R Package Version 1.0.7. https://CRAN.R-project.org/package=factoextra.

[36] R Core Team (2020). R: A Language and Environment for Statistical Computing.

[37] Rohart F., Gautier B., Singh A., Le Cao K.A. (2017). mixOmics: An R package for ‘omics feature selection and multiple data integration. PLoS Comput. Biol..

[38] Bain J. (1958). Morphological, Anatomical, and Physiological Changes in the Developing Fruit of the Valencia Orange, *Citrus sinensis* (L) Osbeck. Aust. J. Bot..

[39] Iglesias D.J., Cercós M., Colmenero-Flores J.M., Naranjo M.A., Ríos G., Carrera E., Ruiz-Rivero O., Lliso I., Morillon R., Tadeo F.R. (2007). Physiology of Citrus Fruiting. Braz. J. Plant Physiol..

[40] Albertini M.-V., Carcouet E., Pailly O., Gambotti C., Luro F., Berti L. (2006). Changes in Organic Acids and Sugars during Early Stages of Development of Acidic and Acidless Citrus Fruit. J. Agric. Food Chem..

[41] Levy Y., Bar-Akiva A., Vaadia Y. (1978). Influence of Irrigation and Environmental Factors on Grapefruit Acidity1. J. Am. Soc. Hortic. Sci..

[42] Castle W.S. (1995). Rootstock as a Fruit Quality Factor in Citrus and Deciduous Tree Crops. N. Zld. J. Crop Hortic. Sci..

[43] Knight T. (2001). The Relationship Between Oil Gland and Fruit Development in Washington Navel Orange (*Citrus sinensis* L. Osbeck). Ann. Bot..

[44] Voo S.S., Grimes H.D., Lange B.M. (2012). Assessing the biosynthetic capabilities of secretory glands in citrus peel. Plant Physiol..

[45] Porat R., Deterre S., Giampaoli P., Plotto A. (2016). Biotechnology in Flavor Production.

[46] Turek C., Stintzing F.C. (2013). Stability of Essential Oils: A Review. Compr. Rev. Food Sci. Food Saf..

[47] Calandra M.J., Impellizzeri J., Wang Y. (2015). An HPLC Method for Hydroperoxides Derived from Limonene and Linalool in Citrus Oils, Using Post-Column Luminol-Mediated Chemiluminescence Detection: An HPLC Method for Limonene and Linalool Hydroperoxides. Flavour Fragr. J..

[48] Dugo G. (2010). Citrus Oils: Composition, Advanced Analytical Techniques, Contaminants, and Biological Activity.

[49] Tùnder D., Petersen M.A., Poll L., Olsen C.E. (1998). Discrimination between freshly made and stored reconstituted orange juice using gc odour profling and aroma values. Food Chem..

[50] Hinterholzer A., Schieberle P. (1998). Identification of the most odour-active volatiles in fresh, hand-extracted juice of valencia late oranges by odour dilution techniques. Flavour Fragr. J..

[51] Buettner A., Schieberle P. (2001). Evaluation of Aroma Differences between Hand-Squeezed Juices from Valencia Late and Navel Oranges by Quantitation of Key Odorants and Flavor Reconstitution Experiments. J. Agric. Food Chem..

[52] Buettner A., Mestres M., Fischer A., Guasch J., Schieberle P. (2003). Evaluation of the Most Odour-Active Compounds in the Peel Oil of Clementines (*Citrus reticulata* Blanco *cv. clementine*). Eur. Food Res. Technol..

[53] Selli S., Kelebek H. (2011). Aromatic profile and odour-activity value of blood orange juices obtained from Moro and Sanguinello (*Citrus sinensis* L. Osbeck). Ind. Crops Prod..

[54] Feng S., Suh J.H., Gmitter F.G., Wang Y. (2018). Differentiation between flavors of sweet orange (*Citrus sinensis*) and mandarin (*Citrus reticulata*). J. Agric. Food Chem..

[55] Elston A., Lin J., Rouseff R. (2005). Determination of the Role of Valencene in Orange Oil as a Direct Contributor to Aroma Quality. Flavour Fragr. J..

[56] Perez-Cacho P.R., Rouseff R.L. (2008). Fresh squeezed orange juice odor: A review. Crit. Rev. Food Sci. Nutr..

[57] Takeoka G.R., Teranishi R., Williams P.J., Kobayashi A. (1996). Characterization of citrus aroma quality by odor threshold values. Biotechnology for Improved Foods and Flavors.

[58] Plotto A., Margaría C.A., Goodner K.L., Goodrich R., Baldwin E.A. (2004). Odour and flavour thresholds for key aroma components in an orange juice matrix: Terpenes and aldehydes. Flavour Fragr. J..

[59] Arena E., Guarrera N., Campisi S., Nicolosiasmundo C. (2006). Comparison of odour active compounds detected by gas-chromatography–olfactometry between hand-squeezed juices from different orange varieties. Food Chem..

[60] Rodríguez A., Peris J.E., Redondo A., Shimada T., Costell E., Carbonell I., Rojas C., Peña L. (2017). Impact of D-limonene synthase up- or down-regulation on sweet orange fruit and juice odor perception. Food Chem..

[61] Dugo G., Verzera A., d’Alcontres I.S., Cotroneo A., Trozzi A., Mondello L. (1994). On the Genuineness of Citrus Essential Oils. Part XLIII. The Composition of the Volatile Fraction of Italian Sweet Orange Oils (*Citrus sinensis* (L.) Osbeck). J. Essent. Oil Res..

[62] Näf R., Velluz A., Meyer A.P. (1996). Volatile Constituents of Blood and Blond Orange Juices: A Comparison. J. Essent. Oil Res..

[63] Wu G.A., Terol J., Ibanez V., López-García A., Pérez-Román E., Borredá C., Domingo C., Tadeo F.R., Carbonell-Caballero J., Alonso R. (2018). Genomics of the Origin and Evolution of Citrus. Nature.

